# Alcohol consumption during pregnancy by women from southern Brazil: a cross-sectional study

**DOI:** 10.1590/1516-3180.2023.0186.R1.08022024

**Published:** 2024-04-22

**Authors:** Laira Francielle Ferreira Zottis, Mateus Arenhardt de Souza, Jéssica Karine Hartmann, Thiago Kenji Kurogi Gama, Laís Borges Rizental, Anita Machado Maciel, Merialine Gresele, Ernani Bohrer da Rosa, Maurício Rouvel Nunes, Juliana Trevisan da Rocha, Jorge Alberto Bianchi Telles, André Campos da Cunha, Paulo Ricardo Gazzola Zen, Rafael Fabiano Machado Rosa

**Affiliations:** I Undergraduate Student, Department of Clinical Medicine, Universidade Federal de Ciências da Saúde de Porto Alegre (UFCSPA), Porto Alegre (RS), Brazil.; II Undergraduate Student, Department of Clinical Medicine, Universidade Federal de Ciências da Saúde de Porto Alegre (UFCSPA), Porto Alegre (RS), Brazil.; III Undergraduate Student, Department of Clinical Medicine, Universidade Federal de Ciências da Saúde de Porto Alegre (UFCSPA), Porto Alegre (RS), Brazil.; IV MD. Physician, Resident Doctor of Anesthesiology, Universidade de São Paulo (USP), São Paulo (SP), Brazil.; V MD. Physician, Resident Doctor of Trauma Surgery, Hospital de Pronto Socorro de Porto Alegre (HPS), Porto Alegre (RS), Brazil.; VI BSc. Biomedical, Clinical Research Data Manager, Santa Casa de Misericórdia de Porto Alegre (SCMPA), Porto Alegre (RS), Brazil.; VII MD. Physician, Resident Doctor of Internal Medicine, Universidade Federal de Ciências da Saúde de Porto Alegre (UFCSPA), Porto Alegre (RS), Brazil.; VIII BSc. Nurse, Doctoral Student, Postgraduate Program in Pathology, Universidade Federal de Ciências da Saúde de Porto Alegre (UFCSPA), Porto Alegre (RS), Brazil.; IX BSc. Nurse, Doctoral Student, Postgraduate Program in Pathology, Universidade Federal de Ciências da Saúde de Porto Alegre (UFCSPA), Porto Alegre (RS), Brazil.; X PhD. Biomedical, Professor, Discipline of Histology and Embryology, Department of Basic Health Sciences, Universidade Federal de Ciências da Saúde de Porto Alegre (UFCSPA), Porto Alegre (RS), Brazil.; XI MSc. Fetologist and Obstetrician, Department of Fetal Medicine, Hospital Materno Infantil Presidente Vargas (HMIPV), Porto Alegre (RS), Brazil.; XII MD. Obstetrician, Department of Fetal Medicine, Hospital Materno Infantil Presidente Vargas (HMIPV), Porto Alegre (RS), Brazil.; XIII PhD. Clinical Geneticist and Pediatrician, Professor, Departments of Clinical Medicine and Clinical Genetics, Universidade Federal de Ciências da Saúde de Porto Alegre (UFCSPA), Porto Alegre (RS), Brazil.; XIV PhD. Clinical Geneticist, Professor, Departments of Clinical Medicine and Clinical Genetics, Universidade Federal de Ciências da Saúde de Porto Alegre (UFCSPA), Porto Alegre (RS), Brazil.

**Keywords:** Alcohol Drinking, Pregnancy, Tobacco Smoking, Risk factors, Pregnancy Outcome, Alcohol Intake, Family Planning, Gestation

## Abstract

**BACKGROUND:**

Some maternal characteristics are related to alcohol intake during pregnancy, which irreversibly compromises the maternal-fetal binomial integrity.

**OBJECTIVES:**

To identify the frequency, impact, and factors associated with alcohol consumption during pregnancy.

**DESIGN AND SETTING:**

A cross-sectional study was performed at the Hospital Materno Infantil Presidente Vargas (HMIPV) in Porto Alegre/RS between March and December 2016.

**METHODS:**

A structured questionnaire was administered along with a medical records review. They refer to the maternal sociodemographic and gestational status, alcohol consumption patterns, and characteristics of the fetus/newborn. In the statistical analysis, P values < 0.05 were considered significant.

**RESULTS:**

The frequency of alcohol intake was 37.3%; this was characterized by the consumption of fermented beverages (89.3%), especially during the first trimester (79.6%). Risky consumption (high and/or early) occurred for 30.2% of participants. Risk factors associated with maternal alcohol consumption during pregnancy were tobacco use (P < 0.001) and abortion attempt (P = 0.023). Living with a partner (P = 0.002) and planning pregnancy (P = 0.009) were protective factors. Risky consumption was related to all of the aforementioned variables as well as threatened abortion (P = 0.023).

**CONCLUSIONS:**

Alcohol intake during pregnancy is common and affects nearly one-third of pregnant women. Knowledge of the population at risk and protective factors is essential for the development of campaigns that seek to reduce consumption and, therefore, its consequences for the mother and fetus.

## INTRODUCTION

Health problems related to alcohol consumption are usually associated with males. However, changes in the social role of women have led to a decrease in this difference.^1^ Over time, a progressive rise in the use and abuse of alcohol by women has been observed,^2^ even at childbearing age, which has led to an increasing number of obstetric and neonatal complications.^3^ According to the World Health Organization (WHO), the global prevalence of alcohol intake is 9.8%.^4^ In Brazil, this reality is even more concerning, with frequencies ranging from 6% to 60%.^5-7^

Some maternal characteristics are associated with an increased frequency and amount of alcohol intake during pregnancy. These include lifestyle, psychological factors, alcohol consumption patterns, concomitant drug use, social vulnerability, and aspects related to family structure, such as single motherhood.^8^ The lack of prenatal care and pregnancy planning also appears to be important predisposing factors.^9,10^

Alcohol can cause injuries in one in every 100 live births exposed during pregnancy,^11^ and there is no safe consumption amount. Therefore, abstinence is the best and safest conduct to be followed.^12^ Alcohol directly acts on fetal and maternal-fetal tissue and, indirectly, on nutrients and oxygen supply, which can result in devastating and irreversible effects on the embryo/fetus.^13^ Consequently, the spectrum of fetal consequences may be wider, ranging from only cognitive and behavioral disorders to fetal alcohol syndrome.^14,15^

Thus, these effects and consequences highlight the importance of early detection and profiling of women at risk of alcohol use during pregnancy. This will enable the development of more effective management and follow-up approaches.

## OBJECTIVE

Considering the scarcity of Brazilian epidemiological studies related to this issue, our aim was to identify the impact and factors associated with alcohol intake in pregnant women who gave birth at a reference hospital in southern Brazil.

## METHODS

This was a prospective cross-sectional study carried out at the Hospital Materno Infantil Presidente Vargas (HMIPV) that included pregnant and postpartum women and their respective newborns who utilized the hospital’s obstetrics service at birth from March to December 2016. All patients were users of the Unified Health System (in Portuguese, Sistema Único de Saúde – SUS), which is the current public health system in Brazil. The HMIPV is located at Porto Alegre and is a reference hospital for maternal and child care in the State of Rio Grande do Sul (RS).

Trained members conducted a pilot study and applied the clinical protocol. Data were collected through direct interviews with puerperal women during rooming in shortly after delivery. The medical records of the mothers and their respective newborns were also reviewed. The data collected included sociodemographic and gestational history findings, fetuse/newborns characteristics, and the use of drugs, including alcohol, during pregnancy. The sample comprised 570 women who were divided into two groups: 1) those who did and 2) did not consume alcohol during pregnancy.

Maternal age was stratified into women aged 20 years or younger, those 21–34 years, and those 35 years or older.^16^ Origin was classified by whether the patient lived in Porto Alegre, metropolitan region, or countryside town of the state. Puerperal women’s education was classified as incomplete elementary education, complete elementary education, and complete secondary education to higher education.^17^ The number of residents per house into two groups: from one to five and from six to 14 individuals.^18^ Family income was categorized into one minimum wage (BRL 880.00 at the moment of the study) or more per household. Women were divided into primiparous or multiparous groups according to their number of pregnancies.^19^ Prenatal care was divided by trimester of beginning (in first, second, or third)^20^ and by consultations number (fewer than six or six or more).^21^

Women were asked about quarter(s) of alcohol consumption; alcohol amount consumed and intensity, frequency, and type of drink. All beverage measurements were converted and presented according to international (10 g of pure alcohol per serving)^22^ and national standards (14 g of pure alcohol per serving). This corresponds to approximately 350 mL of beer, 150 mL of wine, or 45 mL of hard liquor.^2^

Alcohol consumption amount was categorized as low (1–20 g/day), moderate (21–40 g/day) or high (≥ 41 g/day).^23^ Binge drinking episodes and heavy episodic drinking were assessed according to WHO guidelines and defined as the consumption of 60 g or more of pure alcohol, or about four servings or more on at least one occasion.^22^ Drink types were questioned and classified into fermented (beer, sparkling wine, and wine) and distillates (whiskey, vodka, “cachaça,” “caipirinha,” and tequila). Consumption frequency was classified as monthly or less, two to four times a month, two to three times a week, or four or more times a week. Women who answered affirmatively to at least one of the following variables were categorized as engaging in risky consumption: period of use in the first trimester, consumption frequency of four or more times a week, high amount consumption (≥ 41 g/day), or positive for binge drinking episodes.^24^

Prematurity was defined as gestational age at birth less than 37 weeks^25^ and low birth weight as below 2,500 grams.^26^ For Apgar scores, values greater or less than 7 were considered at the first and fifth minutes of life.^27^ When fetal malformations were present, they were classified as isolated or multiple and specified according to the medical records description. In assessing microcephaly, the measurement of the newborns head circumference was considered both for gestational age at birth (absolute) and after correction, considering baby’s length (relative or true).^28^

The results for qualitative variables are presented as frequencies and percentages, and the quantitative variables are shown as averages and standard deviations. Normality was verified using the Shapiro-Wilk test and histogram inspection. Sociodemographic and gestational factors associated with alcohol consumption were evaluated using a Poisson regression analysis with robust variance adjustment. Prevalence ratio (PR) measurements are presented with a 95% confidence interval. For multivariate analysis, variables with P < 0.20 in the Wald test were selected. To evaluate the characteristics of fetuses and live births, Student’s t-test and chi-square tests were applied, as was Fisher’s exact test when appropriate. The results were considered statistically significant at P < 0.05. The analyses were performed using SPSS statistical software (SPSS Inc., Chicago, United States, Release 22.0, 2013).

This study was approved by the Research Ethics Committee of HMIPV on October 10, 2017 (CAAE 09909712.3.1001.5329), and the Universidade Federal de Ciências da Saúde de Porto Alegre (UFCSPA) on January 12, 2018 (CAAE 9909712.3.3001.5345). The study was only performed after informed consent assignment.

## RESULTS

The total sample consisted of 570 women, ages ranging from 12 to 45 years (mean 25.1, SD ± 7.1). Gestation was recognized at an average of 11.6 weeks. Paternal ages ranged from 15 to 60 years (mean 28.5, SD ± 8.5). The average number of maternal pregnancies was 2.32 (range 1–12).

Alcohol consumption was reported by 213 women (37.3%). Age was similar between women who consume and did not consume alcohol (mean age of 24.9 [SD ± 6.8] and 25.2 years [SD ± 7.2] respectively; P = 0.5887). Most women who drank alcohol during pregnancy consumed low amounts (64.2%; moderate: 16.9%; high: 18.9%). However, approximately one in every four women (24.9%) reported binge drinking episodes. Fermented beverages were the main type of beverage consumed (89.3%), with beer representing the most common (82.1%), followed by vodka (17%) and wine (9.2%). Almost 13% of women drank both fermented and distilled beverages during pregnancy. Most drank monthly or less (63.9%; two to four times a month: 27.4%; two to three times a week: 6.7%; and four or more times a week: 1.9%). The first trimester was the most commonly reported period of alcohol use (79.6%), with a decreasing trend in the other trimesters (second trimester: 48.3%; third trimester: 33.6%). Continuous use throughout pregnancy was reported by 21.8% of women.

Table 1 presents the consumption of alcoholic beverages during pregnancy according to maternal sociodemographic characteristics. There was a significant association between presence of a partner and lower alcohol consumption (PR = 0.66; 95%CI: 0.53-0.83; P < 0.001). However, beverage consumption was significant in two situations: when the pregnancy was unplanned (41.9%; PR = 0.72; 95%CI: 0.57-0.91; P = 0.006) and when some abortive intervention was carried out (71.4%; PR = 1.93; 95%CI: 1.20-3.13; P = 0.007; Table 2).

**Table 1.** Consumption of alcoholic beverages according to maternal sociodemographic characteristics

Variables		Total* (n = 570)	Alcohol use (n = 213)	%	P value	PR	95%CI	
		n	n					
Age group	≤ 20 years	183	68	37.2		1		
	21 to 34 years	319	123	38.6	0.757	1.04	0.82	1.31
	≥ 35 years	68	22	32.4	0.489	0.87	0.59	1.29
Origin	Porto Alegre	391	155	39.6		1		
	Metropolitan area	151	49	32.5	0.132	0.82	0.63	1.06
	Countryside town	28	9	32.1	0.456	0.81	0.47	1.41
Employed (n = 568)*	Yes	228	90	39.5		1		
	No	340	123	36.2	0.424	0.92	0.74	1.14
Education	Incomplete elementary education	200	79	39.5		1		
	Complete elementary education	188	69	36.7	0.571	0.93	0.72	1.20
	Complete secondary education to higher education	178	62	34.8	0.351	0.88	0.68	1.15
Presence of partner	No	100	52	52.0		1		
	Yes	470	161	34.3	0.000	0.66	0.53	0.83
Family income (n = 550)*	Less than R$ 880	112	41	36.6		1		
	> R$ 880	438	164	37.4	0.871	1.02	0.78	1.34
Number of residents per house (n = 566)*	< 5 individuals	315	110	34.9		1		
	≥ 5 individuals	251	101	40.2	0.193	1.15	0.93	1.43

Quantitative variables are presented as means and standard deviations in the text

* Number of valid answers; PR = Crude prevalence ratio; CI = Confidence interval.

**Table 2.** Consumption of alcoholic beverages according to gestational characteristics

Variables		Total* (n = 570)	Alcohol use (n = 213)	%	P value	PR	95%CI	
		n	n					
Planned pregnancy	No	351	147	41.9		1		
	Yes	219	66	30.1	0.006	0.72	0.57	0.91
Number of gestations	Multiparous	340	128	37.6		1		
	Primiparous	230	85	37.0	0.867	0.98	0.79	1.22
Attempted abortion	No	563	208	36.9		1		
	Yes	7	5	71.4	0.007	1.93	1.20	3.13
Prenatal care	No	12	6	50.0		1		
	Yes	558	207	37.1	0.310	0.74	0.42	1.32
Number of consultations (n = 555)*	> = 6 consultations	430	157	36.5		1		
	< 6 consultations	125	50	40.0	0.471	1.10	0.85	1.40
Beginning of prenatal care (n = 558)*	1	375	132	35.2		1		
	2	166	71	42.8	0.087	1.22	0.97	1.52
	3	17	4	23.5	0.363	0.67	0.28	1.59
zz	No	518	189	36.5		1		
	Yes	51	24	47.1	0.110	1.29	0.94	1.76

* Number of valid answers; PR = Crude prevalence ratio; CI = Confidence interval.

Drinking during pregnancy was associated with both concomitant use of illicit (PR = 2.20; 95%CI: 1.72-2.80; P < 0.001) and licit drugs (tobacco; PR = 1.96; 95%CI: 1.61-2.39; P < 0.001) as well as use during the first trimester of pregnancy (P < 0.001). Alcohol intake during gestation occurred in 78.3% of women who had used illicit drugs and 59.6% of those who smoked. Approximately three out of four marijuana users had consumed alcohol. Only eight women reported using cocaine (3.8%), and three crack (1.4%). All of them reported using alcohol simultaneously (Table 3).

**Table 3.** Consumption of alcoholic beverages according to concomitant use of drugs during pregnancy

Variables		Total* (n=570)	Alcohol use (n=213)	%	P value	PR	95%CI	
		n	n					
Use of illicit drugs during pregnancy	No	547	195	35.6		1		
	Yes	23	18	78.3	0.000	2.20	1.72	2.80
Use of illicit drugs during 1st trimester of pregnancy	No	548	196	35.8		1		
	Yes	22	17	77.3	0.000	2.16	1.68	2.78
Tobacco during pregnancy	No	434	132	30.4		1		
	Yes	136	81	59.6	0.000	1.96	1.61	2.39
Tobacco 1st trimester	No	437	134	30.7		1		
	Yes	133	79	59.4	0.000	1.94	1.59	2.36
Marijuana	No	550	198	36.0		1		
	Yes	20	15	75.0	0.000	2.08	1.58	2.75
Marijuana 1st trimester (n = 569)*	No	550	198	36.0		1		
	Yes	19	14	73.7	0.000	2.05	1.53	2.74
Cocaine	No	562	205	36.5		1		
	Yes	8	8	100	0.000	2.74	2.46	3.06
Cocaine 1st trimester (n = 569)*	No	562	205	36.5		1		
	Yes	7	7	100	0.000	2.74	2.46	3.06
Crack	No	567	210	37.0		1		
	Yes	3	3	100	0.000	2.70	2.43	3.01
Crack 1st trimester (n = 569)*	No	567	210	37.0		1		
	Yes	2	2	100	0.000	2.70	2.43	3.01

* Number of valid answers; PR = Crude prevalence ratio; CI = Confidence interval.

None fetal or newborn characteristic was associated with consumption of alcoholic beverages by women. However, ‘multiple” malformations were most predominant in the group of fetuses exposed to alcohol (46.2%; Table 4).

**Table 4.** Fetus or newborn characteristics according to maternal consumption of alcoholic beverages during pregnancy

Variables		Total^a^	Alcohol use	%	P value	PR	95%CI	
		n*	n*					
Malformation	No	550	204	37.1				
	Yes	33	13	39.4	0.787	1.06	0.69	1.64
Type of Malformation	Multiple	9	6	66.7		1		
	Isolated	24	7	29.2	0.037	0.44	0.20	0.95
Thoracic	No	580	214	36.9		1		
	Yes	3	3	100.0	0.000	2.71	2.44	3.01
Skeletal	No	580	215	37.1		1		
	Yes	3	2	66.7	0.154	1.80	0.80	4.03
Gastrointestinal	No	580	215	37.1		1		
	Yes	3	2	66.7	0.154	1.80	0.80	4.03
Urinary tract	No	572	212	37.1		1		
	Yes	11	5	45.5	0.542	1.23	0.64	2.36
Extremities	No	578	215	37.2		1		
	Yes	5	2	40.0	0.895	1.08	0.37	3.16
Abdomen	No	580	216	37.2		1		
	Yes	3	1	33.3	0.892	0.90	0.18	4.45
Central nervous system	No	573	215	37.5		1		
	Yes	10	2	20.0	0.322	0.53	0.15	1.85
Other	No	578	215	37.2		1		
	Yes	5	2	40.0	0.895	1.08	0.37	3.16
Prematurity	>=37	502	186	37.1		1		
	<37	73	28	38.4	0.828	1.04	0.76	1.41
Low birth weight	>=2,500g	506	187	37.0		1		
	<2,500g	69	27	39.1	0.723	1.06	0.77	1.45
Absolute microcephaly (n = 489)^a^	No	473	175	37.0		1		
	Yes	16	7	43.8	0.563	1.18	0.67	2.09
Relative microcephaly * (n = 489)^a^	No	482	179	37.1		1		
	Yes	42	16	38.1	0.902	1.03	0.68	1.54
Apgar 1 < 7* (n = 569)^a^	> = 7	518	185	35.7		1		
	< 7	51	25	49.0	0.040	1.37	1.01	1.86
Apgar 5 < 7* (n = 569)^a^	> = 7	562	206	36.7		1		
	< 7	7	4	57.1	0.181	1.56	0.81	2.99

* Relative microcephaly corrected for the length of the baby; Apgar 1<7/Apgar 5<7 = values greater or less than 7 in the first and fifth minutes of life; a. Number of fetuses/neonates, considering deaths and valid responses; PR = Crude prevalence ratio; CI = Confidence interval.

Multivariate analysis showed that trying to terminate the pregnancy increased the chance of alcohol consumption by 63% (PR = 1.63; 95%CI: 1.04-2.54; P = 0.032), while smoking increased it by 90% (PR = 1.90; 95%CI: 1.55-2.32; P < 0.0001; Table 5). However, the presence of protective factors is noteworthy. The likelihood of alcohol consumption for a pregnant woman with a partner was 23% lower than that for women with no partner (PR = 0.77; 95%CI: 0.61-0.97; P = 0.024). In addition, planning the pregnancy reduced the chance of alcohol consumption by one quarter (PR = 0.75; 95%CI: 0.59-0.94; P = 0.013; Table 5).

**Table 5.** Multiple regression analysis of factors associated with alcohol consumption during pregnancy

Variables	P value	PR	95%CI	
Marital status = With partner	0.024	0.77	0.61	0.97
Planned pregnancy = Yes	0.013	0.75	0.59	0.94
Attempted abortion = Yes	0.032	1.63	1.04	2.54
Smoking = Yes	0.000	1.90	1.55	2.32

Valid variables with P < 0.20 in the Wald test were included in the analysis: PR = Adjusted prevalence ratio; CI = Confidence interval.

Risky alcohol consumption was observed in 171 women (30.2%) and was associated with attempt to terminate the pregnancy (PR = 1.92; 95%CI: 1.17-3.13; P = 0.009), threat of abortion (PR = 1.45;95%CI: 1.05-1.99; P = 0.023), and tobacco use (PR = 2.10; 95%CI: 1.66-2.65; P < 0.0001). The presence of a partner (PR = 0.66; 95%CI: 0.51-0.86; P = 0.002) and planned pregnancy (PR = 0.74;95%CI: 0.56-0.97; P = 0.027) were protective factors against risky alcohol consumption (Table 6).

**Table 6.** Multiple regression analysis of factors associated with risky consumption during pregnancy

Variables	P value	PR	95%CI	
Marital status = with partner	0.002	0.66	0.51	0.86
Planned pregnancy = Yes	0.027	0.74	0.56	0.97
Attempted abortion = Yes	0.009	1.92	1.17	3.13
Threat of abortion = Yes	0.023	1.45	1.05	1.99
Smoking = Yes	0.000	2.10	1.66	2.65

Valid variables with P < 0.20 in the Wald test were included in the analysis; Risk consumption considered women who answered affirmatively to at least one of the variables: quantity (high > = 41g/day), frequency (4 or > week), EBP (positive), and consumption in the first trimester; PR = Adjusted prevalence ratio; CI = Confidence interval.

## DISCUSSION

This study identified a high frequency of alcohol consumption in pregnant women (37.3%), similar to other Brazilian studies, such as those that took place in São Paulo (33.3%),^6^ Rio de Janeiro (40.6%),^28^ and Teresina (32.4%).^29^ These frequencies are higher than those reported in developed countries (10.2%).^30^ In addition to methodological and sampling differences, some factors, such as effectiveness of public health policies and cultural disparities, may have contributed to such inequalities.^26^ In general, alcohol consumption in developed countries is seen more negatively than in Latin America.^31^

The results obtained in our study about the use of alcohol in the first trimester of pregnancy and the occurrence of binge drinking episodes among pregnant women were cause for concern. They were similar to those reported by other authors^9,10^ and highlight the progressive tendency of the female population to consume more alcohol at abusive levels.^32^ Early consumption of alcohol within the first trimester has been associated with a 12-fold greater chance of fetal involvement. Moreover, excessive levels of alcohol intake have increased the risk for worse neonatal outcomes.^33^

Alcohol consumption most often occurred in an occasional way and in low quantities; this fact reflects the underestimation of the harmful effects even for small amounts.^11^ In addition, the drinks mainly consisted of fermented beverages, especially beer and derivatives, which may also demonstrate an underestimation of the potential risk of these beverages compared to distilled drinks.^34^

The absence of a partner has been shown to be closely related to alcohol consumption by women during pregnancy.^8,9,13^ According to the Centers for Disease Control and Prevention (CDC), alcohol consumption is almost twice as common among pregnant women with no partner. In addition, the frequency of excessive consumption among these women is significantly higher.^35^ This finding is often related to other risk factors for alcohol consumption, such as unplanned pregnancies, as well as low socioeconomic status, with the mothers usually being the main or only providers of income.^36^ Notably, in the present study, the presence of a partner was a protective factor for alcohol consumption, which may be related to greater financial and emotional stability as well as family support.

Another protective factor identified in this study was pregnancy planning. There are reports in literature of substantially less alcohol consumption by women during pregnancy when there is intention and planning to become pregnant.^9,37^ Failure to plan and, consequently, delay to recognize the pregnancy, can lead many women to engage in harmful behaviors, including the consumption of alcoholic beverages.^10^ This fact helps to clarify the high frequency of alcohol intake (79.6%) during the first trimester observed in our study. In addition, women tend to be more careful about what they consume when pregnancy is planned, as they are more aware of possible risks for the fetus due to alcohol consumption.^38^

The use of pregnancy termination methods was associated with risky alcohol intake in our sample. This association is well described in the literature.^39^ This may be linked to pregnancy rejection because it is unwanted.

Threatened abortion was the only variable that showed an exclusive association with at-risk alcohol consumption in this study. According to the literature,^40^ women who consume more than five drinks per week have a significantly greater chance of miscarriage. This problem occurs because alcohol is one of the main substances responsible for childbirth-related problems.^41^

As previously described in the literature,^5,15,41^ we found that smoking was an important factor associated with risky alcohol consumption. The simultaneous use of alcohol and tobacco can be explained by the legality and wide availability of these substances as well as by the common maternal risk factors for their use during pregnancy, which are usually related to the vulnerability presented by these women.^42^ In addition, studies have identified neurotransmitters and nicotine receptors that interact with both substances and mediate effects involved not only in their sensitivity but also in dependence.^43^

The most discussed and described fetal malformations associated with alcohol consumption belong to the spectrum of findings observed in individuals with FAS.^14^ However, some studies point to possible associations between maternal alcohol consumption and the occurrence of other anomalies.^43^ In the present study, no major fetal/neonatal malformation was associated with the consumption of this substance. Alcohol can cross the placental barrier and directly interfere with embryonic and fetal development. However, there is no single mechanism that can explain all of the harmful effects of alcohol on the fetus or the precise alcohol amount that can cause malformations.^9^

Other factors have been associated with alcohol consumption during pregnancy, such as low education and socioeconomic level, advanced maternal age, and lack of prenatal care.^8,9,13^ However, we did not find significant relationships among them in the present study.

Despite the care we took in terms of both sample size and data collection and analysis, we cannot rule out possible limitations such as memory bias. Previous literature indicates that parents who have children with malformations tend to remember more details about their previous history, such as the use of substances during pregnancy.^44^

## CONCLUSION

Therefore, alcohol intake during pregnancy is common at our hospital. There is also high use during the first trimester as well as preferential consumption of fermented products and in abusive amounts, which represents important cause for concern. This risk of alcohol intake applies mainly to women who smoke, attempt pregnancy termination, or have a threat of miscarriage. However, there are protective factors such as the presence of a partner and pregnancy planning. Considering the unique characteristics of this vulnerable population, these findings may be important in the development of more effective campaigns for the avoidance of alcohol consumption by women during gestation.

Moreover, healthcare professionals should be prepared to identify efficient strategies during prenatal care, given the apparent lack of awareness in a considerable number of situations involving this issue in routine consultations.
